# Observations on the Temperament and Diseases of the People Inhabiting the Highlands of Aberdeenshire

**Published:** 1805-01-01

**Authors:** Tuaam Peaal


### Observations on the Temperament and Diseases of the People inhabiting the Highlands of Aberdeenshire. By Mr. Tuaam Peaal.

FEW are the diseases that rage among the inhabitants of the Highlands; I believe they enjoy a greater share of health than falls to the lot of those of any other nation. This can only be the effect of temperance and regular excitement; the want of effeminacy of manners; moderate exercise; a simple and regular regimen; a pure air; a certain serenity of mind, in being less agitated by the anxious cares of commercial life, which distract the thoughts, and dissipate the animal spirits of those, who live in the lower parts of the country, where traffic is carried on to a much greater extent. Both men and women in the Highlands have a well regulated deportment, which excludes many moral evils or vices that lay the sure foundation from whence proceed a fatal train of physical evils, both incidental and hereditary, that insensibly sap the constitution, and by quick degrees bring the unhappy victims of them to a premature death. In this way I presume their advantages above others, in point of health, may be accounted for. There may be others of a more private and domestic nature, with which we are not so well acquainted, such as the tone and harmony of the passions, whose operations are only seen to advantage in the obscure

---

scure recesses of social life; for it is well known that these have a mighty influence on the animal œconomy, and according to their movements either cherish or destroy the functions of life. In general, I believe, they are in a great measure free from those convulsive operations of passion that enervate the animal frame, by being removed from that luxury and dissipation, that lust of avarice and ambition, which so easily kindle the soul into a flame, and shake its frail companion to the very base on every disappointment it meets with from the jarring collision of opposing interests. On the contrary, by having few desires, and fewer opportunities of gratifying the corrupt propensities of nature, they are consequently exempted from the evils those unsatiable appetites uniformly bring; they may be said to live to nature, and therefore enjoy the advantages she brings to all those who follow her wholesome dictates.

Having premised these introductory remarks, I shall begin first with considering the female sex, whose temperament differs considerably from that of the male, owing to the different conformation of their body, mode of life, and education.—They are of a smaller stature than the male; some of them have a strong muscular system, while others again are thin, and have rigid fibres. In general, they are never corpulent; many of them are very ruddy complexioned, but those that are advanced in years are rather swarthy. Their bones are for the most part strong, and well formed; they abound with a large proportion of blood, and their catamenia usually continue three days. They are very patient under suffering, and are very seldom violently affected with anger, or talk much without a meaning. From want of experience and observation, their reasoning faculties and knowledge must be defective. It is only by being conversant with different objects that our ideas are formed, and unless we be surrounded with objects capable of exciting perfect ideas, our reflexion, the operation of the mind, and the retention of ideas, will be but weak, and therefore the faculty of discerning between ideas comparatively small. This is the reason why their knowledge is so much upon a par, and from the want of ideas, and improvement of the mind, female judgement is not so very perfect as the male, who receives a better education; which draws out to view every latent virtue and perfection which would never make their appearance without such help. They are very susceptible of love, good natured, and bear love slights with the greatest fortitude. They endure the vicissitudes of the climate surprisingly, for for it is no uncommon thing with the women to be going barefooted, and washing their cloaths, when the ground is covered with ice. Some of them are addicted to hysteria; this disease is more frequent however with old maids than young females. In the Highlands, the women when with child take a good deal of exercise, which is a means of preventing sickness, vomiting, and ischuria, so frequent among pregnant females of the city. Abortion is very unfrequent, as is also laborious and preternatural labours. Laborious labours, however, do sometimes occur, and are for the most part owing to the bones of their childrens' heads being very much ossified, and large. Weakly women have often easier labours than the strong and robust. The reason is obvious, the bones of their childrens' heads are not so strong, they more easily mould themselves to the capacity of the pelvis, and the parts are not so tense. The Highland women when with child, are very healthy, and they always nurse their own children. They give them however too long suck, and it is not uncommon with them to continue nursing until they be with child again. But when this does not occur, they will give them suck for one year and a half. They commit another error in giving the child suck too often; this is common with nurses every where. Whether it be owing to their having a pleasure in giving it, to the milk stimulating their breasts to have a discharge, or to still the child's crying, I cannot tell; but it would tend much to the child's health to regulate its instinct in this particular, and to give it suck only at stated times. By giving it frequently the child's stomach becomes gorged with it: he then sickens; which increases until he vomits it. When the mothers think their childrens' stomachs will bear it, they give them milk pottage (milk boiled with oat or barley meal) twice or three times a day, which is a very proper food, when given in moderation. They generally give them plenty of airing, which is of great advantage to children, as it makes them strong and hardy. When the child becomes sickly or unwell their only cure for it is whisky punch, which is also employed when there is a scarcity of the mother's milk. The weaning brash does not occur so often in the Highlands as in the city, where it is very common, and frequently terminates in rickets. When diarrhœa happens to children in the Highlands, the principal cure is boiled milk and eggs, inflammed whisky, or whisky punch, which is also employed in the colic of children. Disorders of the primæ viæ and intestines, form the largest portion of those which affect affect children in the Highlands. The food of children in the Highlands, after they are weaned, does not differ much from what is used by their fathers. It is principally of a vegetable nature. Milk, eggs, butter, and cheese, are however much used in some parts. Corn meal boiled with water into pottage is frequently used for breakfast, dinner, and supper. It is boiled very thick, and supped with milk. Sometimes for dinner greens or kail are used. In ancient times, artemesia was boiled with water, and used for dinner with bread as a substitute for kail. This was a dish which the Highlanders were very fond of, and what agreed well with those who were accustomed to it. For supper, sowns, pottage, milk and bread, or boiled potatoes are often used.

There is no doubt that this simple aliment used by the Highlanders, joined with exercise, produces that particular temperament, which so remarkably characterizes them. They become strong and hardy, but are less irritable and sensible than those who live upon animal food; hence they are less subject to diseases. Too great an irritability or tenderness of the constitution, subjects to disease upon the slightest excess or difficience of the accustomed impressions; this is not the case with the Highlanders, they can bear fatigue, cold, and abstinence from food and drink without throwing them into disease, so that the excitement or vitalment is increased or diminished without affecting the vitality so much as to produce even predisposition.

Until of late years the knowledge of the Highlanders was very much confined, their education consisting chiefly in learning to read; but this reading was not extended to such books as could improve the mind. The Bible, and a few school books, formed all their library; and after they could read, these were only studied at leisure hours. It requires reflection before reading can be a rational amusement to the human soul, and it requires a good deal of reading and application, which those who follow laborious work have seldom time for, before they become acquainted with the learned and illustrious dead, or the enlightened living. But what principally tends to form the temperament of the Highlanders, is the climate, and their pursuits, being almost always without the house, tending cattle, or employed about some rural affairs. This produces in them great muscular force; and as the air is generally pure, and free from noxious exhalations, they are not subject to the diseases arising from impure air.

Although they have not many opportunities of acquiring knowledge, knowledge, or arming themselves with maxims of prudence and reflection, yet their minds are open to improvement, and they have a perfect knowledge, even to scientific precision, of such things as come within their sphere. They have a desire to reflect upon such things as pass under their review, are very social, and seldom quarrelsome; but when once provoked they are very fierce. The colour of their hair is various. We meet with black, umber brown, light umber brown, red, and light red. The colour of the iris of their eyes is also various, being different shades of black, brown, and light blue. Their temperaments are however much changed when they dwell for some time in the city, and arrive at the dignity of a Ballie or Provost. By this we see the difference which a change of eating, drinking, want of exercise, and a more cultivated state of mind, produces on strong natural talents. Instances of longevity are very common in the Highlands, both among the male and female sex; few of them die until they be fourscore, unless they have been much weakened by repeated attacks of disease. And those who chance to remove to the city in their younger years, unless cut off by fever or some contagious disease, live till they be aged sixty years.

It would be beneficial in practice to be acquainted with the different temperaments of individuals, and yet there remains great uncertainty about their diagnosis and arrangement. Two practitioners would give a different name to the temperament of the same individual; which has been the occasion of my giving over mentioning the temperament of the patient when I am drawing up a case.

But some time ago I was led to adopt a different arrangement, by an endeavour to find out the temperament of a reverend reader of sermons not far from Aberdeen, who had a strong muscular system, ill-looking countenance, weak memory and judgment; envious and passionate. The countenance is very often an index of the mind, whatever be the profession. A truly religious and benevolent mind being happy, is always accompanied with an open, free, and cheerful countenance. Professional and studious men are more under the influence of intellectual enjoyment and suffering than any other class. "The man who is benevolent, wise, and courageous, possessing the greatest enjoyment and least suffering, is most happy." Misery is the reverse of this. The effects of each of these states upon the body must be obvious. I have divided the temperaments into four classes.

1. Those

1. Those with a strong muscular system, large bones, and head, without much mental exercise: subject to be affected by those things whose effects depend upon opinion. Of this temperament are most of the laborious, or rustic employment.

2. With a mean degree of muscular strength rather slender, unused to hard labour, tenacious memory; sometimes weak; subject to violent mental opinion; envious; without those graces which constitute the excellence of man. Constituting the majority of the middle class of men; including sometimes professional gentlemen and the clergy.

3. Weak muscular system: subject to violent mental and animal suffering. Among this class are included the sensualist and hypochondriac.

4. Those whose animal and intellectual senses are regulated to form the chief excellence of man; as probity, supported by wisdom, temperance, and fortitude. Of this temperament are many female characters. It is accompanied with a moderate degree of muscular strength, serenity of countenance, and good nature. They possess the greatest enjoyment and least suffering, and are therefore most happy.

All the houses in the Highlands, which are of one story, are built without chimnies; the fire place is at one end of the house, close to the gable wall; the smoke being light, ascends, and passes out at an aperture left in the roof for the purpose; this opening has a small wooden top resembling a chimney; and at one side a piece of board raised above the top of the cubical frame, with a stalk that comes down for the purpose of turning it to the side which the wind comes from, so that the smoke has more liberty to escape; this is a simple contrivance to prevent smoke, and gave origin to the more elegant ones, which turn with the wind. Yet notwithstanding this, when there is much wind, the houses are frequently smoky. Their fuel for fire consists of peat, which is charred vegetables, and consists principally of carbon. The couples and rafters of their houses are covered with an oily bitumen occasioned by the carbonic acid and hydrogen; it very much resembles tar, and has equally as good an effect in preserving the wood from rotting. There is no doubt but the Highlanders inhale the carbonic vapour into their lungs, which would be a drawback upon their health, if it were not for their being so much exposed to the pure air, and their being accustomed to the smoke. The smoke of their houses is very disagreeable to strangers until they be some time used to it. Their fire is placed on the ground, close to the gable of the house, round which they all sit; the heat is very strong, it spreads itself all round, and warms the whole house, which without such large fires would be damp and uncomfortable. It is a very general custom with most of the people in the country, and in the Highlands, to smoke tobacco; and very often, when they associate together, only one pipe is employed by the whole of them through the evening; when one has done smoking, another gets the pipe, and so it goes round the whole company. What will be the consequence if one of those are affected with the sibbens? But there are several diseases which may be caught by the saliva, such as scrophula, putrid sore throat, aphtæ, erysipelas, venereal ulcers, &c. It would be better, if they could not abstain from smoking, that each person should have his own pipe; and not only that, but likewise his own spoon, which would obviate the danger mentioned.

They assert that smoking is good for the tooth-ache, colic, heart-burn, &c. and prevents hunger, fevers, and all kind of infection. But whether it has these good effects I cannot take upon me to determine. It certainly hurts the nervous system, and destroys the appetite, and not unfrequently in some produces a complaint in the stomach which they denominate heart-wringing; smoking generally relieves it, but it returns again, if it be not duly repeated. The practice of smoking, now so general in the Highlands, is a deviation from that natural simplicity of nature so common with them. It is to be lamented that they do not recollect that their forefathers lived without it, and that since its introduction among them, diseases have been more frequent. But custom with them, as well as those of the city, triumphs over reason; and as they live in a more social manner, any bad custom is sooner acquired and propagated than in the city. My remarks on smoking or chewing tobacco, will also apply to the immoderate use of whisky.

In process of time it will be no uncommon thing to see them in the country indulge to great excess in all the luxuries of the city, which will have a great effect in diminishing their bodily strength, and be the cause of many diseases to which they were strangers in their more natural state.

It would be happy for them if they imitated the better sort of people of the city in regard to cleanliness. Indeed, in in this particular they are much reformed, both with respect to their bodies and clothes. The fair sex on Sunday vie with each other in appearing clean and neat, which induces the young men to imitate their example, that they may engross a share of their favour. But in many parts of the Highlands and country, this necessary virtue is much neglected; their skins are covered with a loam of perspirable matter and dust, their beards long, and filled with the same, and their clothes and linen extremely dirty. While this is the case, it is not to be wondered that the itch prevails so much in some parts. For although it depends upon a poisonous virus, which may be denominated soriculi, yet uncleanness increases its virulence and facility of being catched. There is no disease more easily cured. since sulphuric acid has been discovered to be a specific remedy for it. Those are therefore very blameable who allow it to continue on their skins; and many of them are so careless and dirty that they permit the disease to remain, without ever thinking of having it removed, which is an injury both to themselves and neighbours. It is a disorder which gradually spreads over the whole body. The virus enters the interstices of the rete mucosum, raises it into a small pimplar vesicle containing the virus and lymph. A great itching attends, especially when the skin is bare, and exposed to the air; this happens most commonly at bed-time, and is a characteristic mark of the disease. When it continues long upon the skin, it generally produces boils in different parts of the body, and this most commonly in children who are affected with it. I have seen some cases where the feet and head were all in one crust. When it affects children, and allowed to increase without speedy application, it affects, and appears very much like tinea capitis; but in tinea the crust is for the most part white, dry, and thick; this again is more red, and pours out a considerable quantity of thin matter, appears on other parts of the body, and is easier cured than tinea capitis. The itch is a very distressing complaint when it gets into a family where there are a number of young children.

In the city of Aberdeen the itch is less common than in the country, because coals are generally used for fire. Peat or turf in the country. As the coals abound with sulphur they will have a great effect in preventing this disease. I am informed that in the south of Scotland, where sulphury Scots coals are used, that the itch is scarcely known, This

 would would make us conclude that peat fires rather encouraged this disease.

Fixed air has a very great tendency to produce boils; I once had a case of a young man who had a great number of boils upon his body, which he attributed to his drinking too much mineral water of his own making.

The peculiar temperament of some nations induce particular diseases. The Jews had their leprosy. The Americans, syphilis and gonorrhœa. The Africans, frambæsia. The Scots and Irish, the itch. There is not a stronger instance can be urged in favour of cleanliness than that by Moses when he treats of leprosy, which appears, from his description of it, to be a species of frambæsia or yaws; which is a disease very infectious, and more liable to be catched than the itch. Moses describes it with great accuracy, and how it is to be distinguished from inflammation, abscess, and other diseases to which it bears any resemblance. The cure he trusted entirely to cleanliness, washing with water, and shutting the patient up to prevent its contagious influence.

Colic is one of the diseases which we most frequently meet with in the Highlands; it seems to arise from various causes, and to consist of different degrees of violence. Sometimes it yields to a stimulant plan of treatment, which they prescribe for themselves, consisting of black pepper mixed with milk, and sometimes whisky. When it does not yield to this treatment in two days, it is of a more violent nature, and attended with great pain and inflammation, which obliges them to apply for medical assistance. Those cases which I have attended, generally yielded to the same agents that are usually employed for the mill-neck or the painter's colic, as blood-letting, castor oil, fomentations, the warm bath, and injections.

I have attended several cases where the patients were affected with spasms of the intestinal canal of a more chronic nature. The patients would sometimes take a little food, but when in any quantity, it was vomited again along with a mouthful of water. When more than a certain quantity of drink was taken, it was regurgitated in the same manner. They complained of a constant sensation of wind rumbling through their bowels, accompanied sometimes with a sharp cutting pain, a wasting of their body, and great dejection of spirit. These symptoms continued with little variation two or three months, but at last increased to continual hiccup and costiveness, which terminated in death. It would be much better for the Highlanders landers when they drink whisky, to mix it with equal parts of sweet milk, which would obviate its tendency to produce spasms of the bowels, and would make the danger less if any inflammation were present.

Fevers do sometimes occur in the Highlands, but not very frequently; they are generally of that kind called synochus, and occasioned by contagion. You would think that the vigour and hardiness of their bodies would resist its influence, but it is otherwise. When a countryman goes to visit a friend in fever, it is a rare circumstance if he be not affected with it. I have known several countrymen, after they have gone to see their friends in the Aberdeen Hospital, come home affected with fever, which sometimes proves fatal. They are more apt to be infected with fever than those who live in the city, owing to their enjoying the free air. Those in the city are constantly exposed to the effluvia arising from the decomposition of animal substances; they are therefore accustomed to a certain degree of contagion, which renders them not so susceptible of catching fever as those in the country. You observe, in your Essay on Fever,* that there is something in the nature of the contagion of fever which determines it most commonly to produce the same disease. There is always a great similarity between cases of infected fever here; and its action upon the body, in this part of the country, very much resembles cold. Intermittents are entire strangers here.

Inflammation, and soreness of the lips in the cold of winter, and intense heat of the summer, is a common complaint. When this occurs in old subjects, and is neglected or irritated by tobacco pipes when smoking, it sometimes turns into cancer of the lip, a disease very frequent among old men in the Highlands.

We are experiencing the advantage of Dr. Jenner's discovery. Every school-master is becoming a vaccinator. The small-pox can no longer intimidate us. If it do, we have ourselves to blame.

There are a few more diseases which we meet with here, such as stone, hernia, herpes, sibbens, and epilepsy; but I am afraid I have already trespassed upon your patience. I have nothing remarkable to add at present with respect to any of these. The number afflicted with stone in the bladder have decreased one-half, which I suppose is owing to the meal being ground with granite stones. The

 mill-

* This paper was, originally, addressed to Dr. Ferguson. Ed.

mill-stones were formerly very soft, and mouldered fast away in grinding, so that a great part of the meal consisted of earthy and sandy particles.

I have tried your sulphat of soda poultice in two cases of ulcerated bubo, with the same good effect as you mention in chancre. They were cured without applying ointment of any kind.

